# Indirect treatment comparison of dabrafenib plus trametinib versus vemurafenib plus cobimetinib in previously untreated metastatic melanoma patients

**DOI:** 10.1186/s13045-016-0369-8

**Published:** 2017-01-04

**Authors:** Adil Daud, Japinder Gill, Sheily Kamra, Lei Chen, Amit Ahuja

**Affiliations:** 1 Medicine and Dermatology, University of California, 1600 Divisadero Street Rm A 743, San Francisco, CA 94143 USA; 2 PAREXEL International, Chandigarh, India; 3 Novartis Pharmaceuticals Corporation, East Hanover, NJ USA

**Keywords:** Dabrafenib, Trametinib, Vemurafenib, Cobimetinib, Metastatic melanoma, Indirect treatment comparison

## Abstract

**Background:**

Metastatic melanoma is an aggressive form of skin cancer with a high mortality rate and the fastest growing global incidence rate of all malignancies. The introduction of BRAF/MEK inhibitor combinations has yielded significant increases in PFS and OS for melanoma. However, at present, no direct comparisons between different BRAF/MEK combinations have been conducted. In light of this, an indirect treatment comparison was performed between two BRAF/MEK inhibitor combination therapies for metastatic melanoma, dabrafenib plus trametinib and vemurafenib plus cobimetinib, in order to understand the relative efficacy and toxicity profiles of these therapies.

**Methods:**

A systematic literature search identified two randomized trials as suitable for indirect comparison: the coBRIM trial of vemurafenib plus cobimetinib versus vemurafenib and the COMBI-v trial of dabrafenib plus trametinib versus vemurafenib. The comparison followed the method of Bucher et al. and analyzed both efficacy (overall survival [OS], progression-free survival [PFS], and overall response rate [ORR]) and safety outcomes (adverse events [AEs]).

**Results:**

The indirect comparison revealed similar efficacy outcomes between both therapies, with no statistically significant difference between therapies for OS (hazard ratio [HR] 0.94, 95% confidence interval [CI] 0.68 − 1.30), PFS (HR 1.05, 95% CI 0.79 − 1.40), or ORR (risk ratio [RR] 0.90, 95% CI 0.74 − 1.10). Dabrafenib plus trametinib differed significantly from vemurafenib plus cobimetinib with regard to the incidence of treatment-related AE (RR 0.92, 95% CI 0.87 − 0.97), any AE grade ≥3 (RR 0.71, 95% CI 0.60 − 0.85) or dose interruption/modification (RR 0.77, 95% CI 0.60 − 0.99). Several categories of AEs occurred significantly more frequently with vemurafenib plus cobimetinib, while some occurred significantly more frequently with dabrafenib plus trametinib. For severe AEs (grade 3 or above), four occurred significantly more frequently with vemurafenib plus cobimetinib and no severe AE occurred significantly more frequently with dabrafenib plus trametinib.

**Conclusions:**

This indirect treatment comparison suggested that dabrafenib plus trametinib had comparable efficacy to vemurafenib plus cobimetinib but was associated with reduced adverse events.

## Background

Metastatic melanoma is an uncommon but aggressive form of skin cancer, with a high mortality rate [1, 2]. Although melanoma represents less than 5% of all diagnosed skin cancers, the World Health Organization has indicated that its incidence is increasing faster than any other type of malignancy, mainly due to the general population’s increasing exposure to ultraviolet light [3–5]. Estimates put new diagnoses of melanoma at 132,000 globally in 2015 [3, 5]. The year-on-year increase in global incidence of melanoma is estimated to be between 3 and 7%; based on these estimates, thus a doubling in the incidence of melanoma occurs every 10–20 years [3].

Up to 70% of patients diagnosed with melanoma and approximately 50% of patients with the advanced form of melanoma possess a mutation in the BRAF gene, leading to aberrant activation of the mitogen-activated protein kinase (MAPK) pathway, a well-documented cancer pathway [6–9]. Patients with distant metastases and a BRAF mutation have significantly reduced median overall survival (OS) when compared with patients with distant metastases and BRAF wild-type [10]. These attributes have provided the impetus for significant drug development efforts that target BRAF-mutated metastatic melanoma.

The introduction of BRAF inhibitors such as vemurafenib and dabrafenib have yielded significantly improved outcomes in patients with metastatic melanoma with either BRAF V600E or V600K mutations [11, 12]. However, BRAF inhibitors have substantial therapeutic disadvantages. Acquired resistance to such inhibitors frequently develops due to reactivation of the MAPK pathway. This reactivation occurs primarily through three mechanisms: mutations in the upstream RAS proteins, mutant BRAF amplification, and alternative splicing mechanisms [9, 13]. This acquired resistance limits the median progression-free survival (PFS) and OS achievable with BRAF inhibitors to 6–8 months [14, 15]. In addition, the use of BRAF inhibitors may result in the development of secondary skin cancer, further limiting the therapeutic benefit of this monotherapy [11, 13, 16–20].

The addition of a MEK inhibitor along with a BRAF inhibitor can combat the BRAF inhibitor-related resistance and side effects that occur during monotherapy. This combination therapy has demonstrated an increase in median PFS and OS, along with a decrease in the incidence of BRAF-inhibited induced skin tumors [10, 16, 21]. The 2015 United States and European guidelines recommend the use of dabrafenib plus trametinib for metastatic melanoma patients with a BRAF V600 mutation [22, 23]. More recently, the Food and Drug Administration in the United States and European Medicines Agency have approved vemurafenib plus cobimetinib as a combination therapy for patients with BRAF V600E or V600K mutation-positive unresectable or metastatic melanoma [24, 25].

In the absence of evidence from head-to-head trials providing a direct comparison of treatments, health technology assessment agencies require an indirect comparison to help them in their evaluations. In addition, these types of comparisons can inform therapeutic decisions. Srivastava et al. in 2015 published an indirect treatment comparison (ITC) of dabrafenib versus vemurafenib, showing that both dabrafenib and trametinib monotherapies demonstrated comparable PFS and OS, and different tolerability and safety profiles, when indirectly compared with vemurafenib [26].

The objective of this study was to conduct an ITC between two common BRAF/MEK inhibitor combinations, dabrafenib plus trametinib and vemurafenib plus cobimetinib, in patients with metastatic melanoma without prior therapy for the metastatic disease stage in order to further understand the therapeutic and tolerability profile of these therapies.

## Methods

**Fig. 1:**
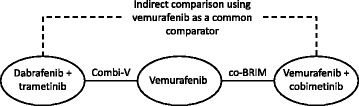
Network diagram for the indirect comparison of trametinib with vemurafenib

A systematic literature review was conducted to identify published studies that would permit an ITC between dabrafenib plus trametinib and vemurafenib plus cobimetinib. From the literature review, two studies were identified that would support an ITC of dabrafenib plus trametinib versus vemurafenib plus cobimetinib using vemurafenib as a common comparator: COMBI-v, an international, open-label, randomized, Phase 3 trial of dabrafenib plus trametinib versus vemurafenib monotherapy in previously untreated patients with unresectable stage IIIC or IV melanoma with BRAF V600E or V600K mutations [27] and coBRIM, an international, multicenter, randomized Phase 3 trial of cobimetinib plus vemurafenib versus vemurafenib plus placebo in previously untreated patients with advanced BRAF-mutated melanoma [21] (Fig. 1). In both trials, vemurafenib 960 mg orally twice daily was administered in the control arm.

The ITC was conducted using the methodology described by Bucher et al. [28]. The method outlined by Bucher et al. in 1997 relies on the principle that the log of the effect size measured for drug A versus drug B is equal to the difference of the log effect size measures for drug A versus drug C and drug B versus drug C [28]. This holds true for both dichotomous outcomes, where risk ratios (RRs) and odds ratios can be used as the effect size measure, and time-to-event outcomes, where hazard ratios (HRs) can be used as the effect size measure. The principal assumption of the model proposed by Bucher et al. is that the relative efficacy of a treatment is the same in all trials included in the indirect comparison, meaning that if A and B are compared in two or more trials, then the effect size of A versus B is similar across all the trials [28]. Further, this method assumes independence between pairwise comparisons, i.e., the comparison of A versus B is independent of the comparison of B versus C [28].

The trials included in the ITC were qualitatively assessed for their patient population in terms of disease characteristics, disease stage, severity of disease, and patient characteristics. Additional subgroup analyses were conducted in cases where a difference in a patient baseline characteristic between the two trial populations was considered potentially clinically meaningful.

The efficacy outcomes that were assessed were overall response rate (ORR), PFS, and OS. The primary efficacy outcome was OS in the COMBI-v trial and PFS in the coBRIM trial. The secondary efficacy outcomes were PFS and ORR in the COMBI-v trial and ORR and OS in the coBRIM trial. Safety was assessed as a secondary endpoint in both of the trials. Multiple data sources including both published and unpublished sources were utilized to retrieve efficacy and safety data for the COMBI-v and coBRIM trials (Table 1).

**Table 1:** Sources and data cut-offs used in primary and additional ITCs

Outcome	COMBI-v		coBRIM	
	Data cut-off	Data source	Data cut-off	Data source
Efficacy outcomes				
OS	Primary Analysis —March 2015 Additional analysis (without crossover)—April 2014 Additional analysis (LDH subgroups)—March 2015	Robert 2015b [29] Robert 2015a [27] Novartis Pharmaceuticals Corporation, unpublished observations	August 2015	Atkinson 2015 [30]
PFS	Primary analysis—March 2015	Robert 2015b [29]	January 2015	Larkin 2015c [32]
	Additional analysis LDH subgroups—April 2014	Novartis Pharmaceuticals Corporation, unpublished observations		
ORR	April 2014	Robert 2015a [27]	January 2015	Larkin 2015c [32]
General adverse events				
Any AE, any SAE, discontinuation due to AE, AE leading to death, any grade ≥3 AE	March 2015	Robert 2015b [29] and Novartis Pharmaceuticals Corporation, unpublished observations	September 2014	EMA label [33, 34]
Any treatment-related AE, any dose interruptions/modifications	April 2014	Robert 2015a [27]		
Specific adverse events				
All specific adverse events except those highlighted in the row below	March 2015	Robert 2015b [29] and Novartis Pharmaceuticals Corporation, unpublished observations	September 2014	EMA label
Keratocanthoma			May 2014	Larkin 2014 [21]
CuSCC—all grades	April 2014			
Chills—all grades grade 3 to 5: alopecia, nausea, pyrexia, vomiting	March 2015		May 2014	EMA label

*AE* Adverse event, *CuSS* cutaneous squamous cell carcinoma, *D + T* dabrafenib plus trametinib, *ECOG* Eastern Cooperative Oncology Group, *EMA* European Medicines Agency, *ITC* Indirect treatment comparison, *LDH* lactate dehydrogenase, *OS* Overall survival, *ORR* Overall response rate, *PFS* Progression-free survival, *SAE* Severe adverse event, *V* vemurafenib, *V + C* vemurafenib plus cobimetinib

The primary ITC for OS and PFS was based on the most recent data cut-off dates; March 2015 for COMBI-v [29] and August 2015 for coBRIM [30] (Table 1). The primary ITC for ORR was based on the April 2014 data cut-off for COMBI-v and the January 2015 data cut-off for coBRIM (Table 1). Of note, crossover was permitted in COMBI-v, following the recommendation by the Independent Data Monitoring Committee (IDMC) based on the planned interim results, whereas no crossover was permitted in coBRIM. The interim analysis was conducted using COMBI-v at the cut-off date of April 2014. To assess whether the crossover might have confounded OS results of the primary ITC, an additional ITC for OS was conducted using the COMBI-v interim data cut-off of April 2014, the point at which no patients had crossed over, and the August 2015 cut-off for coBRIM.

Two other additional ITCs were conducted for OS and PFS in two subgroup populations, i.e., patients with normal and elevated lactate dehydrogenase (LDH) levels, to assess the impact of any variation in the baseline LDH levels on the primary ITC results.

The effect sizes for indirect comparisons were calculated using the methodologies proposed by Bucher et al. [28]. The 95% confidence interval (CI) values and *p* values for the effect sizes were calculated using Cochran-Mantel-Haenszel statistics. All calculations were conducted using STATA® software (version 11).

## Results

The baseline epidemiological and disease characteristics of the patient cohorts in the COMBI-v and coBRIM studies have been reported previously and are summarized in Table 2 [21, 27]. Baseline patient characteristics, including known prognostic factors, were generally well balanced in all the treatment arms of both studies, except that slightly more patients in the coBRIM trial had elevated serum LDH at baseline. Thirty-three percent of patients presented with elevated LDH levels (dabrafenib plus trametinib [34%] and vemurafenib [32%]) in the COMBI-v trial, while the coBRIM trial had 46% of patients with elevated LDH levels (vemurafenib plus cobimetinib [46%] and vemurafenib [43%]).

**Table 2:** Baseline characteristics of patients in the COMBI-v and coBRIM studies

	COMBI-v		coBRIM	
	D + T	V	V + placebo	V + C
Intent-to-treat population	352	352	248	247
Age, median (range), yr	55(18–91)	54(18–88)	55(25–85)	56(23–88)
Male sex, no. (%)	208(59)	180(51)	140(56)	146(59)
ECOG score, no./total no. (%)				
0	248/350(71)	248/352(70)	164/244(67)	184/243(76)
1	102/350(29)	14/352(30)	80/244(33)	58/243(24)
2	0/350	0/352	0/244	1/243(<1)
Metastatic status, no./total no. (%)				
M0	14/351(4)	26/351(7)	13(5)	21(9)
M1a	55/351(16)	50/351(14)	40(16)	40(16)
M1b	61/351(17)	67/351(19)	42(17)	40(16)
M1c	221/351(63)	208/351(59)	153(62)	146(59)
Elevated LDH, no. /total no. (%)	118/351(34)	114/352(32)	104/242(43)	112/242(46)
BRAF mutation, no. /total no. (%)				
V600E	312/346(90)	317/351(90)	174/206(84)	170/194(88)
V600K	34/346(10)	34 /351(10)	32/206(16)	24/194(12)

*D + T* dabrafenib plus trametinib, *ECOG* Eastern Cooperative Oncology Group, *LDH* lactate dehydrogenase, *V* vemurafenib, *V + C* vemurafenib plus cobimetinib

### Efficacy

In the primary ITC, the HR (for OS and PFS) or RR (for ORR) for dabrafenib plus trametinib versus vemurafenib plus cobimetinib was statistically non-significant (Table 3). For OS and PFS, a HR of 0.94 (95% CI 0.68 − 1.30; *p* = 0.7227) and 1.05 (95% CI 0.79 − 1.40; *p* = 0.730), respectively, was observed, while ORR had a RR of 0.90 (95% CI 0.74 − 1.10; *p* = 0.3029) (Table 3). These *p* values and CIs for efficacy outcomes suggested comparable efficacy profiles for the two combination therapies.

**Table 3:** Comparison of efficacy for dabrafenib plus trametinib versus vemurafenib plus cobimetinib

Outcome	COMBI-v		coBRIM		ITC results			
	D + T	V	V	V + C	HR/RR^a^	LCI	UCI	*p* value
Overall survival, median (95% CI), months	25.6(22.6 − NR)	18.0(15.6 − 20.7)	17.4(15.0 − 19.8)	22.3(20.3 − NR)	0.94	0.68	1.30	0.7227
Progression-free survival, median (95% CI), months	12.6(10.7 − 15.5)	7.3(5.8 − 7.8)	7.2(5.6 – 7.5)	12.3(9.5 – 13.4)	1.05	0.79	1.40	0.7300
Overall response rate, no./total no. (%)	226/352(64%)	180/352(51%)	124/248(70%)	172/247(50%)	0.90	0.74	1.10	0.3029

^a^For D + T versus V + C; HR is the output for time-to-event outcomes, i.e., overall survival and progression-free survival; RR is the output for overall response rate

*CI* confidence interval, *D + T* dabrafenib plus trametinib, *HR* hazard ratio, *NR* not reached, *RR* risk ratio, *LCI* lower 95% CI, *UCI* upper 95% CI, *V* vemurafenib, *V + C* vemurafenib plus cobimetinib

To determine if the crossover might have confounded the results of the primary ITC, the additional analysis was conducted using pre-crossover data for COMBI-v (i.e., April 2014 data cut-off) and the August 2015 cut-off for coBRIM. Similar results were shown, i.e., no significant difference in HR between the two combination therapies (HR [95% CI] 0.99 [0.69, 1.41]).

Two other additional subgroup analyses were conducted for OS and PFS outcomes for two subgroup populations, namely, patients with normal and elevated LDH levels at baseline. The ITC results did not show significant differences between dabrafenib plus trametinib and vemurafenib plus cobimetinib for either subgroup in terms of OS (normal LDH levels, *HR* = 0.95 [95% CI 0.58 − 1.54]; elevated LDH levels, *HR* = 1.05 [95% CI 0.67 − 1.65]) and PFS (normal LDH levels, *HR* = 1.05 [95% CI 0.67 − 1.65]; elevated LDH levels, *HR* = 1.23 [95% CI 0.81 − 1.87]).

### Safety

Based on the ITC, the overall toxicity profile of dabrafenib plus trametinib appeared to be better than that of vemurafenib plus cobimetinib. The incidence of any treatment-related adverse event (AE) (RR 0.92, 95% CI 0.87 − 0.97; *p* = 0.0015), incidence of any AE of grade ≥3 (RR 0.71; 95% CI 0.60 − 0.85; *p* = 0.0002), as well as the incidences of dose interruption or dose modification (RR 0.77, 95% CI 0.60 − 0.99; *p* = 0.0471) were all significantly lower with dabrafenib plus trametinib when compared with vemurafenib plus cobimetinib (Table 4). There were no significant differences between dabrafenib plus trametinib and vemurafenib plus cobimetinib with regard to the incidences of any AE (RR 0.98, 95% CI 0.96 − 1.01; *p* = 0.3078), any serious AE (RR 0.84; 95% CI 0.60 − 1.16; *p* = 0.2835), or any AE leading to death or the rate of discontinuation due to an AE (RR 0.62; 95% CI 0.33 − 1.16; *p* = 0.135) (Table 4).

**Table 4:** Comparison of general AEs for dabrafenib plus trametinib versus vemurafenib plus cobimetinib

	Incidence, number (%)				ITC results			
	COMBI-v		coBRIM					
AE type	D + T (*n* = 350)	V (*n* = 349)	V (*n* = 246)	V + C (*n* = 247)	RR	LCI	UCI	*p* value
Any AE	345(98.6)	345(98.9)	240(97.6)	244(98.8)	0.98	0.96	1.01	0.3078
Any serious AE	151(43.1)	136(39.0)	64(26.0)	85(34.4)	0.84	0.60	1.16	0.2835
Any treatment-related AE	320(91.4)	342(98.0)	232(94.3)	237(96.0)	0.92	0.87	0.97	0.0015
AE leading to death	4(1.1)	4(1.2)	3(1.2)	5(2.0)	0.60	0.08	4.35	0.6137
Any grade ≥3 AE	199 (56.9)	232 (66.5)	146 (59.4)	176 (71.3)	0.71	0.60	0.85	0.0002
Any dose interruptions/modifications	192(54.9)	197(56.5)	87(35.4)	110(44.5)	0.77	0.60	1.00	0.0471
Discontinuation due to AE	55(15.7)	48(13.8)	20(8.1)	37(15.0)	0.62	0.33	1.16	0.1350

*AE* adverse event, *CI* confidence interval, *D + T* dabrafenib plus trametinib, *ITC* indirect treatment comparison, *RR* risk ratio, *LCI* lower 95% CI, *UCI* upper 95% CI, *V* vemurafenib, *V + C* vemurafenib plus cobimetinib

With regard to individual AEs, some AEs occurred at a significantly higher incidence with vemurafenib plus cobimetinib compared with dabrafenib plus trametinib, including (in alphabetical order) alopecia, arthralgia, blurred vision, increased blood creatinine, diarrhea, dry skin, dysgeusia, increased alanine transaminase (ALT) and aspartate transaminase (AST), keratosis pliaris, nausea, photosensitivity reaction, pruritus, rash, rash maculopapular, skin papilloma, and sun burn (Table 5). Some AEs occurred more frequently with dabrafenib plus trametinib compared with vemurafenib plus cobimetinib: chills, constipation, cough, and pyrexia.

**Table 5:** Comparison of individual AEs of any grade for dabrafenib plus trametinib versus vemurafenib plus cobimetinib

	Incidence, number (%)				ITC results			
	COMBI-v		coBRIM					
AE type	D + T (*n* = 350)	V (*n* = 349)	V (*n* = 246)	V + C (*n* = 247)	RR	LCI	UCI	*p* value
Abdominal pain	39(11.1)	32(9.2)	19(7.7)	25(10.1)	0.93	0.45	1.91	0.8378
Alopecia	23(6.6)	136(39)	73(29.7)	37(15.0)	0.33	0.19	0.58	0.0001
Anemia	26(7.4)	21(6.0)	20(8.1)	32(13.0)	0.77	0.36	1.67	0.5147
Arthralgia	93(26.6)	182(52.2)	99(40.2)	89(36.0)	0.57	0.42	0.77	0.0003
Asthenia	61(17.4)	58(16.6)	40(16.3)	43(17.4)	0.98	0.59	1.63	0.9368
Blurred vision	17(4.9)	18(5.2)	6(2.4)	25(10.1)	0.23	0.08	0.67	0.0075
Increased blood creatinine	15(4.3)	37(10.6)	20(8.1)	34(13.8)	0.24	0.11	0.52	0.0003
Chills	116(33.1)	28(8.0)	12(5.0)^a^	20(7.9)^#^	2.63	1.19	5.82	0.0167
Chorioretinopathy	2(<1)	1(<1)	1(<1)	31(12.6)	0.06	0.00	1.45	0.0843
Constipation	54(15.4)	25(7.2)	26(10.6)	24(9.7)	2.34	1.17	4.68	0.0160
Cough	77(22.0)	40(11.5)	30(12.2)	19(7.7)	3.04	1.59	5.83	0.0008
Cutaneous squamous cell carcinoma	5(1.4)	63(18.1)	27(11.3)^a^	7(2.8)^#^	0.32	0.10	1.09	0.0685
Decreased appetite	44(12.6)	70(20.1)	50(20.3)	46(18.6)	0.68	0.42	1.13	0.1361
Dermatitis acneiform	23(6.6)	20(5.7)	22(8.9)	34(13.8)	0.75	0.34	1.61	0.4539
Diarrhea	120(34.3)	136(39.0)	82(33.3)	150(60.7)	0.48	0.36	0.64	<0.0001
Dry skin	33(9.4)	67(19.2)	39(15.9)	35(14.2)	0.55	0.31	0.97	0.0407
Dysgeusia	23(6.6)	47(13.5)	26(10.6)	37(15.0)	0.34	0.18	0.67	0.0018
Edema peripheral	48(13.7)	42(12.0)	28(11.4)	31(12.6)	1.03	0.56	1.91	0.9165
Erythema	35(10.0)	42(12.0)	33(13.4)	24(9.7)	1.15	0.60	2.20	0.6795
Fatigue	110(31.4)	117(33.5)	80(32.5)	85(34.4)	0.89	0.64	1.23	0.4697
Headache	112(32.0)	84(24.1)	39(15.9)	41(16.6)	1.27	0.80	2.03	0.3172
Hyperkeratosis	18(5.1)	89(25.5)	75(30.5)	27(10.9)	0.56	0.30	1.06	0.0734
Hypertension	103(29.4)	82(23.5)	19(7.7)	37(15.0)	0.65	0.36	1.15	0.1399
Increased ALT	49(14.0)	61(17.5)	44(17.9)	65(26.3)	0.54	0.34	0.88	0.013
Increased AST	42(12.0)	46(13.2)	31(12.6)	60(24.3)	0.47	0.27	0.82	0.008
Increased blood ALP	26(7.4)	30(8.6)	22(8.9)	36(14.6)	0.53	0.26	1.08	0.0799
Increased blood CPK	10(2.9)	2(<1)	7(2.9)	80(32.4)	0.44	0.08	2.37	0.3378
Increased GGT	38(10.9)	33(9.5)	44(17.9)	54(21.9)	0.94	0.53	1.66	0.8292
Keratocanthoma	2(<1)	35(10.0)	20(8.4)^a^	2(<1)^#^	0.61	0.08	4.58	0.6269
Keratosis pilaris	4(1.1)	48(13.8)	26(10.6)	8(3.2)	0.27	0.08	0.97	0.0442
Myalgia	66(18.8)	56(16.1)	30(12.2)	28(11.3)	1.26	0.71	2.26	0.4298
Nausea	126(36.0)	130(37.3)	62(25.2)	102(41.3)	0.59	0.43	0.82	0.0015
Pain in extremity	45(12.9)	44(12.6)	35(14.2)	24(9.7)	1.49	0.80	2.79	0.2079
Photosensitivity reaction	15(4.3)	81(23.2)	93(37.8)	118(47.8)	0.15	0.08	0.26	<0.0001
Pruritus	36(10.3)	78(22.4)	46(18.7)	48(19.4)	0.44	0.26	0.74	0.0020
Pyrexia	193(55.1)	74(21.2)	56(22.8)	69(27.9)	2.12	1.45	3.09	0.0001
Rash	84(24.0)	150(43.0)	94(38.2)	98(39.7)	0.54	0.39	0.74	0.0001
Rash maculopapular	13(3.7)	28(8.0)	38(15.5)	38(15.4)	0.46	0.22	1.00	0.0490
SCC of skin	2(<1)	21(6.0)	31(12.6)	8(3.2)	0.37	0.07	1.88	0.2310
Skin papilloma	8(2.3)	82(23.5)	29(11.8)	12(4.9)	0.24	0.09	0.62	0.0033
Sun burn	3(<1)	51(14.6)	43(17.5)	34(13.8)	0.07	0.02	0.25	<0.0001
Vomiting	107(30.6)	55(15.8)	31(12.6)	60(24.3)	1.01	0.62	1.64	0.9798

^a^ *N* = 239; ^#^ *N* = 254

*AE* adverse event, *ALP* alkaline phosphatase, *ALT* alanine aminotransferase, *AST* aspartate aminotransferase, *CI* confidence interval, *CPK* creatine phosphokinase, *GGT* gamma-glutamyltransferase, *LCI* lower 95% CI, *RR* risk ratio, *SCC* squamous cell carcinoma, *UCI* upper 95% CI

Focusing on individual AEs that were graded as severe (grade 3 or above), the incidence was in many cases similar between dabrafenib plus trametinib and vemurafenib plus cobimetinib (Table 6). A few AEs occurred more frequently with vemurafenib plus cobimetinib than with dabrafenib plus trametinib: increased ALT, increased AST, rash maculopapular, and rash.

**Table 6:** Comparison of individual AEs of >grade 3 for dabrafenib plus trametinib versus vemurafenib plus cobimetinib

	Incidence (% patients)				ITC results			
	COMBI-v		coBRIM					
AE type	D + T (*n* = 350)	V (*n* = 349)	V (*n* = 239)	V + T (*n* = 254)	RR	LCI	UCI	*p* value
Alopecia	0(0)	1(<1)	1(<1)	1(<1)	0.35	0.01	24.22	0.6295
Anemia	7(2.0)	4(1.2)	6(2.4)	4(1.6)	2.63	0.46	15.10	0.2787
Arthralgia	3(<1)	15(4.3)	12(4.9)	6(2.4)	0.40	0.08	1.91	0.2513
Asthenia	5(1.4)	4(1.2)	3(1.2)	5(2.0)	0.75	0.11	5.17	0.7711
Cutaneous squamous cell carcinoma	5(1.4)	62(17.8)	27(11.3)	6(2.4)	0.38	0.11	1.34	0.1337
Dermatitis acneiform	0(0)	4(1.2)	3(1.2)	6(2.4)	0.06	0.00	1.40	0.0792
Diarrhea	4(1.1)	2(<1)	2(<1)	16(6.5)	0.25	0.03	2.34	0.2242
Fatigue	4(1.1)	7(2.0)	7(2.9)	10(4.1)	0.40	0.09	1.88	0.2459
Hyperkeratosis	0(0)	2(<1)	6(2.4)	0(0)	2.60	0.04	169.50	0.6534
Hypertension	54(15.4)	33(9.5)	6(2.4)	11(4.5)	0.89	0.31	2.58	0.8353
Increased ALT	9(2.6)	15(4.3)	15(6.1)	28(11.3)	0.32	0.12	0.88	0.0280
Increased AST	5(1.4)	9(2.6)	5(2.0)	21(8.5)	0.13	0.03	0.56	0.0062
Increased blood ALP	7(2.0)	5(1.4)	4(1.6)	10(4.1)	0.56	0.11	2.82	0.4826
Increased blood CPK	6(1.7)	1(<1)	0(0)	28(11.3)	0.11	0.00	3.49	0.2076
Increased GGT	19(5.4)	17(4.9)	25(10.2)	32(13.0)	0.87	0.39	1.96	0.7436
Keratocanthoma	2(<1)	35(10.0)	20(8.1)	3(1.2)	0.38	0.06	2.44	0.3091
Maculopapular rash	2(<1)	13(3.7)	13(5.3)	17(6.9)	0.12	0.02	0.61	0.0105
Myalgia	0(0)	4(1.2)	6(2.4)	1(<1)	0.67	0.02	24.45	0.8258
Nausea	1(<1)	1(<1)	2(<1)	2(<1)	0.53	0.02	11.65	0.6871
Pain in extremity	4(1.1)	2(<1)	6(2.4)	3(1.2)	4.00	0.45	35.40	0.2121
Photosensitivity reaction	0(0)	1(<1)	0(0)	7(2.8)	0.02	0.00	1.62	0.0820
Pyrexia	16(4.6)	2(<1)	0(0)	4(1.6)	0.94	0.04	24.60	0.9712
Rash	3(<1)	30(8.6)	14(5.7)	13(5.3)	0.11	0.03	0.43	0.0017
SCC of skin	2(<1)	20(5.7)	31(12.6)	7(2.8)	0.44	0.08	2.32	0.3349
Vomiting	4(1.1)	3(<1)	3(1.3)	3(1.2)	0.94	0.09	9.60	0.9598

*AE* adverse event, *ALP* alkaline phosphatase, *ALT* alanine aminotransferase, *AST* aspartate aminotransferase, *CI* confidence interval, *CPK* creatine phosphokinase, *GGT* gamma-glutamyltransferase, *LCI* lower 95% CI, *RR* risk ratio, *SCC* squamous cell carcinoma, *UCI* upper 95% CI

## Discussion

We applied an established methodology [28] to a simple network based on two trials that were similar in terms of patient populations and trial protocol. The comparison indicated that dabrafenib plus trametinib had comparable efficacy as vemurafenib plus cobimetinib in patients with BRAF-mutated metastatic melanoma, with no statistically significant difference in ORR, PFS, and OS. Comparison of the two combination therapies in terms of AEs found that dabrafenib plus trametinib was associated with a better safety profile and a lower occurrence of AEs. A wide variety of individual AEs occurred more frequently with vemurafenib plus cobimetinib, while fewer occurred more frequently with dabrafenib plus trametinib. When individual severe AEs (grade 3 or above) were compared between treatments, a few occurred more frequently with vemurafenib plus cobimetinib compared with dabrafenib plus trametinib (*p* < 0.05), and no severe AE was observed to occur more frequently with dabrafenib plus trametinib.

The time-to-event outcomes including OS and PFS were evaluated using the most recent data cut-offs of both COMBI-v and coBRIM with different follow-up duration. Specifically, the duration of follow-up was 19 months for dabrafenib plus trametinib and 15 months for vemurafenib in COMBI-v, and 20.6 months for vemurafenib plus cobimetinib and 16.6 months for vemurafenib in coBRIM. Based on the proportional hazards assumption, it can be assumed that variation in the follow-up times between the COMBI-v and coBRIM trials had no significant effect on the results.

The COMBI-v trial allowed crossover following IDMC recommendation, which might have been a confounding factor for the primary ITC. An additional ITC that was conducted using pre-crossover data for COMBI-v (April 2014 data cut-off) and the August 2015 cut-off for coBRIM showed no difference in OS between the two combination therapies, suggesting minimal impact of crossover on the primary ITC.

There was some variation in baseline LDH level between two trials, i.e., COMBI-v had slightly lower proportion of patients with elevated LDH than coBRIM (i.e., COMBI-v: 34% with dabrafenib plus trametinib and 32% with vemurafenib; coBRIM: 46% with vemurafenib plus cobimetinib and 43% with vemurafenib). The additional analyses conducted for OS and PFS outcomes in two subgroup populations, namely patients with normal and elevated LDH levels, showed similar results as the primary ITC in overall population. Specifically, there were no significant differences between dabrafenib plus trametinib and vemurafenib plus cobimetinib for OS and PFS within the subgroup populations defined according to the baseline LDH levels, suggesting that the overall results were not confounded by variation in the distribution of baseline LDH levels.

The advantage of following the method outlined by Bucher et al. [28] is that the randomization of the individual studies is partially retained. Nonetheless, the evidence provided by an ITC is not as strong as that provided by a direct randomized head-to-head trial between the two treatments; the evidence should be considered with this in mind and interpreted with caution. Additionally, concern has been expressed about the suitability of using ITC to compare safety data, due to their non-dichotomous nature. However, it should be noted that health technology agencies such as the French Haute Autorité de Santé and the Agency for Healthcare Research and Quality recommend the use of Bucher’s ITC method for both efficacy and safety outcomes. Additionally, one study has been published using this method when indirectly comparing safety outcomes [31].

Consideration should also be given to the fact that while the ITC indicates a better safety profile of dabrafenib plus trametinib in terms of the frequency of adverse events, this may not translate into a real-world patient experience, where, for example, certain grade 2 adverse events may have a greater impact on a patient’s quality life than a grade 3-elevated AST/ALT. The safety results presented in this manuscript should therefore be interpreted with caution until data from a direct head-to-head trial can provide further insights for physicians. Additionally, COMBI-v and co BRIM trials had different levels of dose interruptions/modifications this may have impacted on the severity of toxicity profiles experienced in both studies.

## Conclusions

In conclusion, in the absence of direct, head-to-head treatment comparisons, ITCs such as the one conducted in this study provided useful information for physicians when evaluating available options of BRAF/MEK inhibitor combinations in order to choose the most suitable treatment for patients, albeit with an understanding of the limitations of such an analysis. The ITC that compared dabrafenib plus trametinib with vemurafenib plus cobimetinib suggested similar efficacies between two combination therapies but reduced adverse events associated with dabrafenib plus trametinib.

## Abbreviations

AE: Adverse event
ALT: Alanine transaminase
AST: Aspartate aminotransferase
CI: Confidence interval
GGT: Gamma-glutamyltransferase
HR: Hazard ratio
ITC: Indirect treatment comparison
LDH: Lactate dehydrogenase
MAPK: Mitogen-activated protein kinase
ORR: Overall response rate
OS: Overall survival
PFS: Progression-free survival
RR: Risk ratio
